# Yeast-Derived Postbiotics for Prevention of Enteric Diseases in Farm Animals: Current Insights and Future Perspectives

**DOI:** 10.3390/vetsci13030287

**Published:** 2026-03-19

**Authors:** Michelle Cerdán-Alduán, Yadira Pastor, Raquel Conde-Álvarez

**Affiliations:** Department of Microbiology and Parasitology, Navarra Medical Research Institute (IdiSNA), University of Navarra, 31008 Pamplona, Spain; mcerdan.3@alumni.unav.es (M.C.-A.); ypastor@alumni.unav.es (Y.P.)

**Keywords:** yeast, postbiotic, livestock, enteric disease

## Abstract

Enteric diseases are a leading cause of morbidity and economic loss in livestock production, and the search for effective, antibiotic-free alternatives has intensified in recent years. Among emerging strategies, yeast-derived postbiotics—non-viable microbial cells and their metabolites—have gained attention for their potential to enhance gut health and disease resistance in farm animals. This review synthesizes current knowledge on the composition, mechanisms of action, and practical applications of yeast postbiotics, particularly those derived from *Saccharomyces cerevisiae* and related species. Key bioactive components, such as β-glucans, mannan oligosaccharides, peptides, and organic acids, are discussed in the context of their immunomodulatory, anti-inflammatory, and pathogen-inhibitory properties. Evidence from in vivo and in vitro studies across multiple livestock species—including poultry, swine, and ruminants—demonstrates beneficial effects on intestinal barrier function, microbial balance, and performance under disease-challenged conditions. Despite promising outcomes, challenges remain in standardizing postbiotic preparations, elucidating dose–response relationships, and tailoring applications to species-specific needs and production systems. This review highlights the potential of yeast postbiotics as a sustainable tool in enteric disease management and outlines research priorities for their broader implementation in animal agriculture.

## Simple Summary

Antimicrobial resistance represents a growing global issue, particularly in livestock, where enteric infections caused by multidrug-resistant bacteria threaten both animal health and farm productivity. Postbiotics offer a safe and promising alternative to manage these infections. Specifically, yeast-derived postbiotics contain a variety of bioactive compounds that support gut health through immunomodulation, inhibition of pathogens, and strengthening of intestinal barrier function. This review provides an overview of current research on yeast-derived postbiotics and highlights their potential role in promoting sustainable livestock health, while identifying challenges and areas for future investigation.

## 1. Introduction

Over the past few decades, global demand for livestock has increased steadily, driven by population growth, urbanization, and rising incomes. This upward trend is expected to continue over the coming years, with estimates suggesting that global demand for milk and dairy products will increase by approximately 63% and 30%, respectively, by 2050 [1]. Overall, the consumption of animal-derived products is expected to rise from 1.4 to 2 billion tons by mid-century [1]. Given the global relevance of livestock products, health challenges in livestock populations can have substantial impacts on food security, public health, and market stability [2]. Recent livestock epidemics highlight these vulnerabilities, such as the avian influenza (H5N1) outbreak between 2014 and 2015, resulting in the cull of 50 million birds and $3.3 billion losses in the poultry industry [3]. Among the most prevalent conditions affecting livestock are enteric diseases, caused by a range of pathogens including *Salmonella*, *Escherichia coli*, *Campylobacter*, *Lawsonia*, *Brachyspira*, *Cryptosporidium*, rotavirus and coronavirus [4,5].

These infections represent a major challenge to livestock production systems, as they increase morbidity and mortality rates, reduce productivity, and ultimately result in substantial economic losses, while also posing risks to food safety and human health [6]. Indeed, frequent and close interactions between humans and animals facilitate zoonotic transmission and heighten the risk of spillover into human populations [7]. This burden is especially evident in low-income regions, where approximately one in seven livestock keepers is affected by zoonotic diseases [8]. Additionally, pathogen exchange at the wildlife–livestock interface represents a critical pathway for disease transmission and the emergence of novel infectious agents [1,9].

Current preventive approaches for these infections encompass improvements in animal husbandry practices, such as reduced stocking density, increased feeder space and removal of soiled bedding [10] or vaccination of either dams or their neonates [11]. However, the effectiveness of these approaches may be limited, and they are often insufficient to achieve effective disease control [12,13,14,15,16,17].

The treatment of these enteric infections is commonly approached through the use of antibiotics. However, the excessive and inappropriate usage of these agents has contributed to the emergence and dissemination of antimicrobial resistance (AMR) in farm animals, leading to the spread of multidrug-resistant bacteria [18,19]. Among the unsustainable and potentially harmful practices in livestock production is the use of antibiotics for growth promotion to increase animal size, in the treatment of persistent infections or even as a preventative measure against disease, which may significantly contribute to the emergence and spread of AMR [1,20]. Moreover, antibiotic use can disrupt the gut microbial balance, impair immune function, and ultimately increase susceptibility to other infections [18,19]. Addressing this growing threat requires a coordinated One Health approach that integrates animal, human, and environmental health to preserve antibiotic efficacy and ensure sustainable livestock production [20].

In this context, many researchers have highlighted the importance of maintaining animal gut health for optimal growth performance, disease resistance, and overall product quality [21]. Thus, considering the detrimental effects of antibiotics on the gut microbiota of livestock, probiotics have emerged as a promising alternative to restore microbial balance and support a more modern and sustainable livestock production system [21,22]. In fact, the global probiotic market is projected to grow from USD 5.5 billion in 2025 to USD 8.27 billion by 2030 [23]. Currently, the most commonly studied and applied probiotics are bacterial strains such as *Lactobacillus*, *Bifidobacterium*, and *Diplococcus* [21,22].

However, the use of bacterial probiotics carries different drawbacks, including their sensitivity to environmental factors such as pH, temperature, and oxygen exposure which can reduce their shelf-life, as well as the potential risk of horizontal transfer of antimicrobial resistance genes to pathogenic bacteria [21,24]. Moreover, recent research has highlighted the potential of some probiotics to produce harmful substances, such as toxins, invasive factors or biofilms [25]. Probiotics may also involve extrinsic challenges related to regulatory approval, public perception, and host-specific administration [25]. In this context, fungal species—particularly yeasts—represent an alternative to bacterial probiotics. Although less extensively studied, yeasts offer several advantages over bacteria: they display greater resistance to gastrointestinal enzymes, bile salts, and variations in pH and temperature. These characteristics contribute to their probiotic potential, including the enhancement of adaptive immune responses and the strengthening of local mucosal protective mechanisms. Nevertheless, live yeasts may also exhibit AMR due to inherent differences in cell wall structure and ribosomal composition, or they may develop tolerance to antifungal agents following exposure, potentially reducing their therapeutic efficacy [21].

Importantly, the adoption of probiotics remains limited among low-income farmers in developing countries due to their relatively high cost [26]. The production and maintenance of high-quality probiotic formulations are expensive, creating a financial barrier that disproportionately affects resource-poor farmers operating on narrow profit margins, who often prioritize short-term survival over long-term productivity gains [26,27]. In regions such as South and Southeast Asia, where antibiotic misuse is widespread, probiotics struggle to compete with inexpensive or substandard antimicrobials that are readily available over the counter [26,27].

In light of these challenges, postbiotics have gained attention as a safer and more stable alternative. Comprising non-viable microorganisms and/or their bioactive metabolites, postbiotics are non-toxic, stable, and capable of providing consistent health benefits. They promote the growth of beneficial bacteria, inhibit pathogenic species within the gastrointestinal microbiome, and have been reported to exert anti-inflammatory, antioxidant, and immunomodulatory effects [28].

Despite the growing interest in yeast-derived postbiotics for livestock production, the available evidence remains fragmented, with inconsistencies in terminology, experimental approaches, dosage regimens, and characterization of postbiotic preparations. These limitations hinder direct comparison among studies and restrict their practical translation. Therefore, the objective of this review is to critically synthesize the current knowledge on yeast-derived postbiotics and their applications in livestock, identify existing gaps, and provide a structured perspective to guide future research and practical application within a One Health context.

## 2. Yeast-Derived Postbiotics

### 2.1. Types of Yeast-Derived Postbiotics

Prior to the consensus definition proposed by the International Scientific Association of Probiotics and Prebiotics (ISAPP), several alternative definitions of postbiotics had been presented in the literature. However, many of these earlier descriptions were considered inadequate because they were vague, inconsistently framed, or imposed arbitrary and impractical criteria. For example, some definitions restricted postbiotics to metabolites derived from probiotics while failing to clearly require a demonstrated health benefit or to consider translational and regulatory implications [29,30]. Consequently, a more precise and standardized definition became necessary [31].

In response to this need, ISAPP defined postbiotics as “a preparation of non-viable microorganisms and/or their components that confers a health benefit on the host” [29,32]. This definition emphasizes that postbiotics consist of microbial preparations in which the microorganisms are no longer viable but may still exert beneficial biological effects [29].

**Figure 1:**
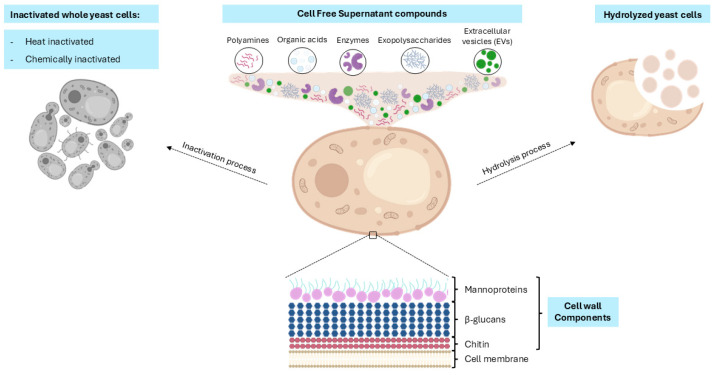
Types of yeast-derived postbiotics. Yeast-derived postbiotics include whole yeast cells inactivated by heat or chemical treatments; molecules released by the cell, such as cell-free supernatant compounds (polyamines, organic acids or enzymes), exopolysaccharides and extracellular vesicles (EVs), hydrolyzed yeast cells obtained through degradation process and structural cell wall components (β-glucans, mannoproteins and chitin). Created in BioRender. Michelle Cerdán (2026). https://app.biorender.com/account/profile (accessed on 15 March 2026).

According to this framework, postbiotic preparations may include different types of microbial-derived material, ranging from inactivated whole microorganisms and intact cells to structural fragments such as cell wall components. In addition, these preparations may contain microbe-derived substances, including metabolites, proteins, or peptides, which may contribute to the overall health effects associated with postbiotics (Figure 1) [30].

Importantly, the ISAPP definition also establishes specific criteria for their characterization and production. A postbiotic must be derived from a well-defined microorganism, or a defined combination of microorganisms, with known genomic sequences. Furthermore, it must be produced through a clearly delineated and reproducible technological process that includes both biomass production and microbial inactivation [30].

However, despite the efforts of the ISAPP to standardize the concept, controversy surrounding the term persists, and a consensus in the literature has yet to be reached. For instance, some researchers consider cell-free supernatants derived from probiotic fermentation as postbiotics, whereas others regard heat-inactivated probiotic cells free of supernatants as postbiotics [32].

The growing interest in postbiotics over recent years is largely driven by their potential health benefits, as well as their favorable safety profile and greater stability compared with preparations containing live microorganisms [29,32].

Currently, several commercial postbiotic products are available, most of which are derived from bacterial strains. These include formulations based on inactivated single bacteria, such as *Bifidobacterium bifidum* MIMBb75; *Lacticaseibacillus paracasei* MCC1849; lysates of *Lactobacillus sakei* proBio65; and pasteurized *Akkermansia muciniphila* or *Vitreoscilla filiformis* or a combination of strains, including *Bifidobacterium breve* C50 and *Streptococcus thermophilus* 065; *Limosilactobacillus fermentum* CNCM MA65/4E-1b and *Lactobacillus delbrueckii* subsp. *delbrueckii* CNCM MA65/4E-2z [31,33]. In contrast, fungal-derived postbiotics have been less explored, and currently only five commercial products are available: one based on a spray-dried, inactivated strain of *Aspergillus oryzae* including its fermentation metabolites (AO), two based on a heat-inactivated strain of *Saccharomyces cerevisiae* (*S. cerevisiae*) (EpiCor^®^ and celluTEIN^®^), one consisting of a β-1,3/1,6-glucan complex extracted from the *S. cerevisiae* cell wall (ABB C1^®^) and one based on a combination of three tyndallized yeast strains: *Saccharomyces boulardii*, *S. cerevisiae*, and *Kluyveromyces marxianus* (ABB C22^®^) [31,34,35,36,37,38]. Although *S. cerevisiae* remains the most studied strain nowadays, in recent years, increasing attention has been directed toward the postbiotic potential of other yeast species, such as *Debaryomyces hansenii*, *K. marxianus*, and *Pichia kudriavzevii* [32].

The available yeast postbiotics comprise structural cell wall components, bioactive metabolites cells or hydrolysis products that support host health and microbiome function. The biological activity of *S. cerevisiae* var. *boulardii* is largely attributed to both its structural components, including β-glucans, mannoproteins, and chitin, and its diverse metabolites, such as polyamines, organic acids, and enzymes [39].

Following an ‘outside–in’ approach, products released into the extracellular environment can be identified, namely cell-free supernatants containing bioactive compounds secreted by the cells (Figure 1). Supernatants obtained from *S. cerevisiae* and *S. boulardii* have been reported to counteract stress-induced alterations in intestinal peristalsis, exert anti-inflammatory and antioxidant effects, and enhance wound healing and intestinal barrier regeneration [40].

Interestingly, extracellular vesicles (EVs) have also gained importance as postbiotic products. Numerous microorganisms, including yeasts, release EVs as a strategy for communication with their environment [41]. These membrane-bound vesicles carry a diverse array of bioactive molecules, such as nucleic acids, proteins, and lipids [42]. Notably, EVs preserve specific traits of their donor cells, incorporating the three major components of the yeast cell wall into their structure [42]. The complexity of their molecular cargo underlies their ability to modulate immune responses. Moreover, their physicochemical properties and functional versatility confer substantial therapeutic potential, positioning EVs as promising tools for biomarker discovery, vaccine development, and targeted drug delivery [41,42]. However, yeast-derived EVs represent an emerging research area, and key aspects remain to be explored, such as their composition, secretion, mechanisms of action, isolation and purification, discovery of novel yeast strains producing EVs, and in vitro and in vivo assessment [43].

Recently, yeast exopolysaccharides have attracted considerable attention due to their health benefits for the host. This complex union of polysaccharides is secreted by yeast cells interacting with the gut microbiota, modulating immune responses and exerting anti-inflammatory effects (Figure 1) [44].

Yeast cell wall components represent another category of postbiotics. Specifically, β-glucans and mannoproteins have demonstrated significant effects on the intestinal immune response by enhancing immunocompetence and promoting beneficial shifts in the host microbiota (Figure 1) [45]. They reduce the abundance of pathogenic bacteria in the ileum and colon, improve intestinal morphology, and consequently enhance growth performance in pigs [45]. Furthermore, both cell wall carbohydrates have been reported to enhance the immune response, with β-glucans promoting proinflammatory cytokine production [46] and mannoproteins strengthening innate and adaptive immunity [47]. The yeast cell wall also contains chitin, which has been shown to possess immunomodulatory properties by activating both innate and adaptive immune responses [48].

Yeast cells can also be hydrolyzed, degrading the cells in a variety of soluble bioactive components (Figure 1). The obtention of hydrolyzed cells can be achieved via autolysis or enzymatic degradation. The first process relies on the activation of intracellular enzymes to solubilize the cell components within the cell, while enzymatic hydrolysis relies on different digestive enzymes to break the cell wall. Thereby, hydrolyzed yeast postbiotics consist of the total array of residual yeast components generated during cell lysis [49]. This category of postbiotics has been shown to enhance overall animal performance, increase milk yield, improve mammary gland health, promote ruminal fiber digestion, and reduce ruminal acetate concentrations [50,51,52].

Another approach to using whole yeast cells as postbiotics involves their inactivation through heat treatment (Figure 1). Various protocols have been described in the literature, employing temperatures ranging from 70 °C to 121 °C, depending on the species and time, to ensure the complete loss of cellular viability [38,53,54,55]. This category of postbiotics is relatively straightforward to produce and can be readily incorporated into diverse food matrices [53]. Moreover, these preparations exhibit notable immunomodulatory activity and have been reported to contribute to the control of foodborne pathogens as well as to the attenuation of symptoms associated with their infection [38,53,54,55].

Additionally, a novel inactivation strategy has been applied to yeast cells through the use of a chemical agent which was previously described for bacterial inactivation (Figure 1). This chemical agent consists of the combination of binary ethylenimine (BEI) with formaldehyde (FA). Yeast postbiotics generated through this protocol have demonstrated the capacity to agglutinate a range of enteric pathogens [55]. Although other chemicals such as hydrogen peroxide, methanol, ethanol, 1-propanol, 2-propanol, acetone, or high-pressure carbon dioxide have been used to inactivate yeast cells [56,57,58] to our knowledge, no research has been found on the postbiotic properties or the safety aspects of these inactivated yeast cells.

### 2.2. Isolation and Purification of Yeast-Derived Postbiotics

According to the recent literature, postbiotics must be intentionally produced, thoroughly characterized, and demonstrated to exert health benefits in the host. Protocols described for obtaining yeast-derived postbiotics are often tailored and time-consuming and require expensive equipment or specialized techniques [59].

The production of postbiotics typically involves the initial selection of a microorganism with the ability to produce bioactive or health-promoting compounds. During the cultivation step, optimized conditions such as pH, temperature, nutrient concentration, and incubation time are required to obtain the desired postbiotic. It is important to take into account that these cultivation parameters not only influence the yield but can also modulate the functional properties and bioactivity of the resulting postbiotics, affecting their applicability across different fields [59]. Subsequently, microbial inactivation is performed using different methods such as thermal treatment, high-pressure processing, UV irradiation, sonication, enzymatic hydrolysis, cold plasma, or CO_2_ extraction. Further concentration steps, including centrifugation, ultracentrifugation or membrane filtration, are employed to separate bioactive compounds from microbial cells. Finally, the obtained postbiotic material undergoes thorough characterization, and quality control assays are needed, to ensure consistency, efficacy and safety. Techniques commonly used for this purpose include high-performance liquid chromatography, gas chromatography–mass spectrometry, and nuclear magnetic resonance [59].

Remarkably, these protocols are primarily standardized at the laboratory scale, and significant challenges arise when scaling up to industrial production (see also Section 5). Therefore, a clear gap exists in knowledge regarding effective yield optimization, which is essential for maximizing the potential of postbiotics in health and industry, driving innovation, and ensuring both cost-effectiveness and compliance with regulatory standards while maintaining product quality [59,60]. Moreover, no harmonized methodology currently exists for postbiotic production, inactivation, or characterization across studies, complicating both the comparison of results and regulatory evaluation [32].

## 3. Mode of Action of Yeast-Derived Postbiotics

**Figure 2:**
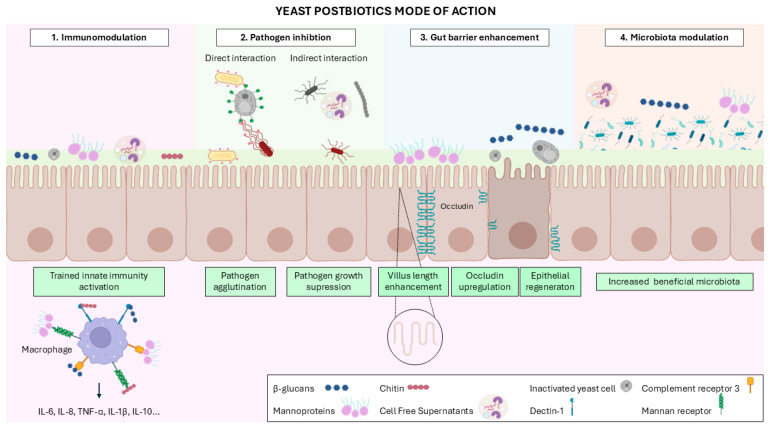
Mode of action of yeast-derived postbiotics. Yeast postbiotics exert their effects through four major mechanisms: 1. immunomodulation, 2. pathogen inhibition, 3. gut barrier and 4. microbiota modulation. Immunomodulatory effects are mediated by recognition through pattern recognition receptors (PRRs), triggering trained immunity, the release of cytokines and consequent activation of immune response. Pathogen inhibition occurs via direct interactions, such as agglutination of pathogens by inactivated yeast cells, and indirect mechanisms, including suppression of pathogen growth. Yeast postbiotics can also promote gut barrier function by enhancing epithelial regeneration, increasing villus length, and upregulating tight junction proteins such as occludins. Finally, yeast postbiotics can support a healthy gut microbiota by promoting the growth of beneficial microbial species. Created in BioRender. Michelle Cerdán (2026). https://app.biorender.com/account/profile (accessed on 15 March 2026).

The health effects of yeast-derived postbiotics can be elucidated through a multifactorial approach assessing their immunomodulatory activity, inhibition of pathogenic microorganisms, effects on gut barrier integrity, and capacity to modulate the composition and function of the gut microbiota (Figure 2).

### 3.1. Immunomodulatory Potential

The immunomodulatory potential of yeast postbiotics is largely driven by their cell wall components. Among these, β-glucans have been largely explored, owing to their well-documented capacity to modulate immune responses. These β-glucans consist of β-1,3-glucan backbones with shorter β-1,6-linked branches. Their numerous hydroxyl groups allow them to adopt a wide range of conformations, including single, triple, or irregular helices, which in turn influence their functional properties [61].

Furthermore, these linkages favor the interaction with pattern recognition receptors (PRRs) such as Dectin-1, complement receptor 3 (CR3), lactosylceramide, Toll-Like Receptors (TLR) 2, 4, and 6, cluster of differentiation 36 (CD36) and scavenger receptors like CD5 [62]. Among these, Dectin-1 and CR3 are well recognized for directly binding β-glucans and thereby modulating immune responses, whereas the remaining receptors primarily participate in downstream signaling cascades [61,62].

More specifically, the activation of the Dectin-1 receptor triggers the activation of two different signaling pathways, Syk kinase and Raf-1, which promote the production of inflammatory cytokines, including TNF, CXC-chemokine ligand 2 (CXCL2), interleukin-2 (IL-2), and IL-10 (Figure 2) [63]. Moreover, this PRR cooperates with TLRs to enhance the release of cytokines such as TNF-α, IL-6, IL-10, and IL-23, while concurrently downregulating IL-12 production [63]. On the other hand, CR3 activation by glucans prepares leukocytes for the cytotoxicity and phagocytosis of target cells [64]. As a result, T and B lymphocytes are activated and so is the adaptive immune response [65]. Additionally, the interaction of yeast β-glucans with CR3 elicits a response similar to that triggered by Dectin-1 engagement, activating PI3K and MAPK signaling pathways and thereby enhancing the production of inflammatory mediators such as TNF-α and monocyte chemoattractant protein-1 [66,67].

Recently, the concept of ‘trained innate immunity’ has been described, referring to the induction of a non-specific immunological memory [68]. This trained immunity can be triggered following β-glucan administration, which activates immune and metabolic pathways and drives epigenetic reprogramming, including methylation and acetylation of promoters and enhancers. These changes strengthen host cellular defense and ultimately promote survival [68]. An in vitro study with human macrophages showed that Dectin-1 activation by β-glucans induced the production of proinflammatory cytokines, including IL-6, IL-8, and TNF-α, through co-engagement with TLR2 and TLR4 [69]. Consistently, an ex vivo study with human macrophages demonstrated that β-glucan stimulation elicits trained innate immunity by increasing the secretion of IL-6 and IL-1β [70]. Indeed, these effects have also been observed in animal cells. A study using porcine monocyte-derived dendritic cells stimulated with β-glucan reported increased expression levels of IL-1β, IL-6, IL-10, and IL-12/IL-23p40 [71]. Similarly, another study conducted in head-kidney leukocytes derived from the Pacific red snapper (*Lutjanus peru*) demonstrated that β-glucan treatment induced the release of IL-1β, TNF-α, IL-6, IL-8 and IL-12 [72]. Additionally, recent research utilized an ovine ruminal explant, in which the ruminal mucosa was isolated and cultured. This mucosa was subsequently exposed to β-glucan, resulting in upregulated gene and protein expression of IL-6 and IL-10 [73].

A similar phenomenon occurs with mannoproteins, which are located in the outermost layer of the yeast cell wall and consist of highly glycosylated proteins. These proteins can be classified into two groups according to the type of glycosylation: N-glycosylated and O-glycosylated. N-glycosylated proteins contain a main carbohydrate chain with multiple branched side units, which is linked to the protein via a N-glycosidic bond. In contrast, O-glycosylated mannoproteins possess a short and unbranched carbohydrate chain. The structure and composition of mannoproteins vary depending on the yeast species, which in turn influences their functional properties [74]. These molecules are readily recognized by PRRs such as CR3, the mannan receptor (MR) or Dectin-2 expressed on innate immune cells [74,75,76]. This interaction triggers the activation of innate immune pathways, leading to the subsequent release of inflammatory cytokines [74,75]. Emerging research has demonstrated that yeast mannans can induce trained immunity in monocytes by augmenting the secretion of TNF-α and IL-6 [76].

Chitin, the third major component of the yeast cell wall, is a linear polymer of β-1,4–N-acetylglucosamine. Along with β-glucans and mannoproteins, chitin functions as a conserved microbial-associated molecular pattern (MAMP) that is recognized by the host immune cells. Its sensing by PRRs, including Dectin-1, MR and TLR-2, initiates proinflammatory immune responses, leading to the production of IL-17, IL-18, IL-23, TNF-α, and LTB4 [45]. This component has also been shown to promote innate immune memory in monocytes by intensifying the production of TNF-α and IL-6 upon a subsequent encounter with the pathogen [77]. Conversely, while numerous studies have demonstrated a proinflammatory role for chitin, other evidence suggests that chitin may also exert immunosuppressive effects by downregulating IL-1β and IL-6 and upregulating IL-10 [78,79,80].

### 3.2. Pathogen Inhibition

Currently, non-antibiotic-based alternatives such as essential oils are commonly employed in the livestock industry to control enteric microbial infections [81]. However, these compounds present several limitations, including chemical instability, potential adverse effects on feed palatability, and variable efficacy under field conditions [82]. In view of these challenges, yeast cells have emerged as a promising alternative, as they are able to directly bind pathogenic microorganisms, thereby preventing or reducing infection. Multiple studies have shown that yeasts can interact with enteric pathogens through the mannoproteins present in their cell wall, which bind to type I fimbriae on pathogenic bacteria [55]. Several yeast strains have demonstrated the ability to agglutinate bacterial pathogens such as Enterotoxigenic *Escherichia coli* (ETEC), *Salmonella enterica* serovar Typhimurium or *Salmonella enterica* serovar Enteritidis (Figure 2) [55,83]. More recently, Hosseini et al. demonstrated that cell-free supernatants containing yeast postbiotics had a potent antibacterial action in vitro and in food models (whole milk and ground meat), showing that supernatants from *S. cerevisiae* effectively inhibited the growth of *Listeria monocytogenes*, *Streptococcus mutans*, *Salmonella typhi*, and *E. coli* [84]. This antibacterial effect is believed to arise from the ability of yeast-derived postbiotics to inhibit the production of essential bacterial enzymes and/or to damage the bacterial cell wall [84].

### 3.3. Gut Barrier Integrity

Yeast postbiotics also exert a direct effect on gut health, which can be assessed using two key indicators: the integrity of the gut barrier and the balance of the gut microbiome. Gut barrier integrity can be compromised by the disruption of tight junctions between enterocytes, which adversely affects its protective functions [85]. The components of the yeast cell wall have been extensively studied for their effects on gut barrier integrity, highlighting their functional relevance [85]. In this sense, in a study evaluating the potential of mannans administered to broiler chicks, the formulation was shown to enhance the expression of tight junction proteins in the ileum, including occluding, following an *E. coli* challenge [85,86]. These findings highlight the ability of mannans to alleviate *E. coli*-induced inflammation and barrier dysfunction [85,86]. Similarly, another study in piglets supplemented with mannans reported improvements in gut barrier integrity, evidenced by enhanced jejunal villus morphology, and immune modulation, with a reduction in TNF-α expression [85,87]. These findings showed beneficial modulation of both structural and immune parameters, associated with enhanced gut health, increased productivity, and improved feed efficiency [85,87].

The commercial postbiotic ABB C22^®^ has been shown to promote gut epithelial development and barrier reinforcement, as well as to confer protection against rotavirus infection. This formulation comprises a combination of three tyndallized yeast strains: *S. boulardii*, *S. cerevisiae*, and *K. marxianus*. In comparative assessments, ABB C22^®^ elicited a stronger and more sustained stimulatory effect on the formation of the digestive epithelium than that observed with a control *S. cerevisiae* strain [38]. To evaluate epithelial integrity and maturation over time, transepithelial electrical resistance (TEER) measurements were conducted across intestinal epithelial cell monolayers, enabling the evaluation of barrier function, paracellular permeability, and tight junction dynamics. The TEER assay revealed a significant and prolonged increase in epithelial barrier resistance in cells treated with ABB C22^®^, indicating enhanced epithelial development and reinforcement [38]. Furthermore, an in vitro assay conducted in rotavirus-infected Caco-2 previously stimulated with ABB C22^®^ showed a reduction in the expression of selected rotavirus genes. Collectively, these results indicate that ABB C22^®^ could potentially contribute to the improvement of intestinal barrier alterations associated with infectious diarrhea and other gastrointestinal conditions [38].

Additionally, β-glucans also play a significant role in preserving gut barrier integrity. A study evaluating ABB C1^®^, a commercial postbiotic composed of a β-1,3/1,6-glucan complex extracted from the *S. cerevisiae* cell wall, reported notable effects on gut epithelial restoration [37]. A TEER assay was conducted in Caco-2 cells treated with ABB C1^®^ postbiotic, to evaluate epithelial integrity. ABB C1^®^ enhanced epithelial regeneration following disruption induced by an ETEC challenge, with improvements that were both pronounced and sustained over time [37]. These results suggest that ABB C1^®^ supplementation could be a valuable strategy to mitigate symptoms and improve outcomes in clinical settings [37].

### 3.4. Microbiota Modulation and Mycotoxin Adsorption

Maintaining a balanced microbiome also influences intestinal defense systems through multiple mechanisms. Postbiotics can modulate the gut microbiota through the diverse range of bioactive molecules they contain, exerting both direct and indirect effects. Directly, postbiotics may exhibit antimicrobial activity against pathogenic microorganisms, thereby contributing to colonization resistance. Indirectly, they provide metabolites and other molecules that can be utilized by different members of the gastrointestinal microbial community, influencing microbial composition and metabolic activity [29,85]. Mannans have shown the ability to promote the proliferation of beneficial bacteria genus, such as *Bacteroides*, *Lactobacilli* and *Bifidobacterium,* in both human and buffalo models [85]. In another research project conducted in high fat diet-induced obese mice model, mannoproteins increased the alpha diversity of the gut microbiota and shifted its overall community structure toward that observed in mice fed a normal diet [88].

Alongside mannans, β-glucans have also demonstrated a role in the restoration of the gut microbiota. In a mouse model of loperamide-induced constipation, glucans derived from baker’s yeast effectively restored gut microbiota composition to levels similar to those observed in the control group [89]. An additional in vivo investigation in pigs administered β-glucans from *Laminaria digitata*, *Laminaria hyperborea* and *S. cerevisiae* demonstrated that these glucans promoted a more diverse community of *Lactobacillus* [90].

In addition to isolated β-glucans, complex yeast-derived postbiotic formulations have also been shown to modulate the gut microbiome. EpiCor, a commercial *S. cerevisiae*-based postbiotic containing bioactive molecules such as vitamins, polyphenols, phospholipids, and cell wall-derived polysaccharides including β-glucans and mannans, has also been shown to modulate microbial composition. Continuous administration of this postbiotic for one week increased the abundance of *Bifidobacterium*, enhanced interactions between *Actinobacteria* and *Firmicutes,* and consequently promoted higher butyrate production [91].

Beyond their effects on the gut microbiota, yeast-derived postbiotics have emerged as a promising antioxidant strategy. Oxidative stress can compromise cellular proteins, membrane lipids, and DNA, leading to damage that may become irreversible and contribute to the development of various diseases, including cancer, cardiovascular disorders, and diabetes. Consequently, antioxidants represent a critical therapeutic and preventive strategy for mitigating the detrimental effects associated with oxidative stress. Specifically, mannans obtained from *S. cerevisiae* have demonstrated effective free radical scavenging activity in the DPPH (2,2-diphenyl-1-picrylhydrazyl) assay. Additionally, mannan-based postbiotics derived from *Candida utilis*, *S. cerevisiae*, and *Candida albicans* have exhibited both antioxidant and antimutagenic properties, with those produced from *C. utilis* showing the most pronounced effects [85].

Moreover, bioactive compounds such as cell-free yeast supernatants have demonstrated superior antioxidant properties compared with live intact cells. This enhanced antioxidant capacity is attributed to the diverse array of active molecules present in the supernatants—including β-glucans, α-mannans, mannoproteins, lipids, and cell wall proteins—which effectively scavenge free radicals [92]. In fact, β-glucans effectively scavenges hydroxyl radicals and singlet oxygen, safeguarding DNA against oxidative damage. Furthermore, in vivo studies employing these polymers in mice with adjuvant-induced arthritis demonstrated a reduction in plasma carbonyl levels, indicating an attenuation of the oxidative tissue damage associated with the progression of arthritic disease [93].

In addition to their role as antioxidants, yeast-derived postbiotics can be fermented by the gut microbiota into bioactive compounds, such as short-chain fatty acids (SCFAs), which play essential roles in both intestinal and systemic health. Notably, mannoproteins derived from *S. cerevisiae* have been able to increase acetate production and activate G protein-coupled receptors, a mechanism that may contribute to the prevention of obesity [88]. A similar effect has been reported for zymosan, a β-glucan-based postbiotic, which enhanced overall SCFA production. Specifically, levels of acetic acid, propionic acid, CO_2_, and H_2_ increased significantly, whereas the production of isovaleric acid and NH_3_ was markedly reduced [94].

Finally, yeast-derived postbiotics have also been associated with the mitigation of mycotoxin-related toxicity. Mycotoxins are secondary metabolites produced by fungi of the genera *Aspergillus*, *Penicillium*, and *Fusarium*. These compounds pose significant public health risks due to their widespread occurrence and their toxic effects in both humans and animals. Exposure to mycotoxins can lead to immunotoxicity, neurotoxicity, hepatotoxicity, nephrotoxicity, reproductive and developmental toxicity, as well as carcinogenic effects. Three yeast cell wall-derived postbiotics—including yeast cell wall, yeast cell wall extract, and a postbiotic yeast cell wall-based blend—were evaluated in vitro to assess their efficacy in sequestering various mycotoxins commonly found in ruminant feed and feedstuffs that may exert adverse effects on the bovine mammary gland, including beauvericin, deoxynivalenol, citrinin and ochratoxin A. The results showed that the postbiotics were able to adsorb the tested mycotoxins to varying degrees. Notably, the efficacy of these adsorbents was associated with a reduction in cytotoxicity [95].

## 4. Applications in Livestock

**Figure 3:**
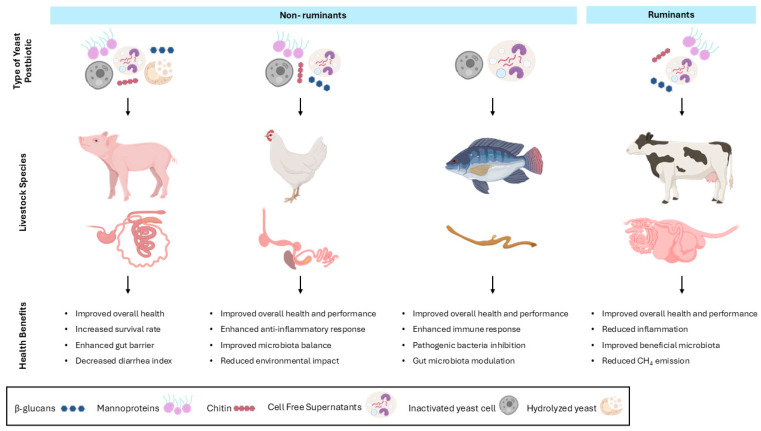
Schematic representation of the different types of yeast-derived postbiotics according to animal species with distinct digestive tract characteristics and their main health benefits. Specific references for each study are provided in Table 1. Created in BioRender. Michelle Cerdán (2026). https://app.biorender.com/account/profile (accessed on 15 March 2026).

As previously noted, the livestock industry represents one of the largest sectors worldwide, underscoring the need for sustainable management practices essential to enhance animal productivity and ensure quality for healthy, antibiotic-free, and sustainable foods [28]. In this context, postbiotics have emerged as a promising alternative to both probiotic and feed antibiotics in animal production systems [28,96]. The use of antibiotics is increasingly discouraged or forbidden due to the rise of antibiotic-resistant bacteria. In this sense, postbiotics offer a safer and more stable alternative compared to probiotics, as they consist of non-living microbial components, meaning the absence of risks associated with the horizontal transfer of antibiotic resistance genes [28,96]. Consequently, postbiotics have been promoted as functional feed additives for monogastric livestock, particularly poultry and swine, to support animal health, growth and overall production efficiency [28,96]. In this section, we review the main studies that have used yeast-derived postbiotics in livestock (see also Table 1 and Figure 3).

### 4.1. Porcine Applications

A growing body of research has evaluated the effects of various yeast-derived postbiotics on the health of sows and their piglets. In fact, they show that dietary supplementation with yeast postbiotics derived from *Pichia guilliermondii*, *S. cerevisiae* fermentation-derived postbiotics (SCFPs), inactivated cells of *S. cerevisae* or yeast fermentation metabolites increases backfat deposition in late gestation, improves lactation efficiency, enhances piglet survival and growth, reduces mortality and diarrhea, increases the number of piglets weaned, and enables earlier weaning [97,98,99,100]. Regarding gut microbiota, the supplementation of sows from late gestation with yeast-derived postbiotics through lactation has been shown to increase the Chao1 index (a species richness estimator) and α-diversity of fecal microorganisms during lactation, enhance the abundance of *Actinobacteria* and *Limosilactobacilli* in sow feces during pregnancy, and promote the presence of beneficial bacteria such as *Bacteroidetes* in piglet feces, thereby improving intestinal health [101]. Moreover, concerning gut integrity, another study reported that administering SCFPs to sows upregulates the expression of tight junction proteins, such us claudin-1 and occludin, indicating enhanced intestinal barrier function [98].

Post-weaning diarrhea (PWD) is a common condition in sows and represents a major challenge in swine production, as it adversely affects growth, increases morbidity and compromises piglet health, causing substantial economic losses in the swine industry [102]. This disease, caused by ETEC, typically appears within two weeks after weaning and is characterized by sudden death or acute diarrhea, dehydration, and growth retardation in surviving piglets [102]. It was traditionally treated with colistin; however, due to the emergence of AMR in recent years, experts have recommended reducing its use [102]. As a result, new non-antibiotic strategies have been developed to control PWD, including nutritional supplements such as yeast postbiotics, which aim to enhance intestinal integrity and reduce the incidence of gastrointestinal diseases in livestock [102].

Within this framework, several in vivo studies have been conducted to elucidate the role of yeast-derived postbiotics during the post-weaning period. Consistently, weaned piglets administered with SCFP and heat-treated *P. kudriavzevii*-derived postbiotics exhibited reduced diarrhea incidence, improved growth rates, and enhanced intestinal health, with a significant enrichment of beneficial bacteria, particularly *Lactobacillus* spp. [103,104]. Notably, during ETEC infection, SCFP postbiotic treatment led to bacterial growth retardation, ameliorated intestinal damage, and reduced diarrheal symptoms by maintaining redox and immune homeostasis through the modulation of key gut microbiota [103]. Interestingly, this immune response was attenuated by yeast postbiotics, as levels of proinflammatory cytokines such as IL-6, TNF-α, and IFN-γ were decreased, whereas anti-inflammatory cytokines (IL-4 and IL-10) were raised in both serum and jejunal mucosa [103].

Finally, yeast postbiotics may also benefit piglets by acting as mycotoxin adsorbents. An in vivo study showed that a mycotoxin detoxifier composed of clay (bentonite and sepiolite), phytogenic feed additives (curcumin and silymarin), and yeast-derived postbiotics improved redox status, exhibited potential hepatoprotective effects, and enhanced growth performance in weaner piglets [105].

### 4.2. Poultry Applications

The poultry industry faces challenges comparable to those in pig production, with various enteric diseases representing significant threats. Among these, necrotic enteritis (NE), coccidiosis, and salmonellosis are the most prevalent and frequently interact, resulting in high mortality rates and considerable production losses. Moreover, these three diseases remain difficult to treat, as the antimicrobials traditionally used are now limited due to the emergence of antibiotic resistance [106,107,108,109]. Once again, the emphasis on substituting antimicrobial treatments relies on finding novel alternatives [110].

It has been proposed that the administration of basal diets supplemented with mannans, SCFP and *S. cerevisiae*-derived postbiotics to broilers enhances overall health and performance, as evidenced by increases in average daily feed intake, average daily gain (ADG), body weight, body weight gain, improvements in feed conversion efficiency, and reductions in cholesterol and corticosterone levels [111,112,113]. Regarding gut health, broilers treated with SCFP and *S. cerevisiae*-derived postbiotics exhibited a significant increase in villus height, villus-height-to-crypt-depth ratio, and numbers of *Lactobacillus* spp. in the cecum [112,113]. Additionally, SCFP elicited a significant reduction in the counts of total *E. coli*, enterohaemorrhagic *E. coli*, ESBL-producing *Enterobacteriaceae*, and *Salmonella* [112]. Positive effects on the immune response were also observed following the administration of *S. cerevisiae*-derived postbiotics, including increased expression of proinflammatory cytokines such as IL-6, nuclear factor-κB, and IL-1β [113].

Another in ovo study, in which the *S. cerevisiae*-derived fermented compounds were directly injected into the fertilized egg and followed through post-hatch, demonstrated that supplementation with this postbiotic has the potential to enhance chick performance, mitigate pathological detriments associated with NE, and positively modulate the mRNA expression of key nutrient transporters during NE challenge [114].

In addition, pullets challenged with *S. enteritidis* and supplemented with *S. cerevisiae*-derived postbiotics showed a reduction in the pathogen colonization in the ceca by 4.49–3.35 log CFU/g compared to the positive control [113]. Furthermore, indirectly exposed pullets treated with SCFP exhibited lower cecal *S. enteritidis* loads and a reduced number of birds testing positive for *S. enteritidis* over time [115]. Broiler chicks dispensed with SCFP revealed reduced stress susceptibility by reducing the heterophil-to-lymphocyte ratio and plasma corticosterone and improved feed conversion [116].

### 4.3. Ruminant Applications

The ruminant industry, which comprises cattle, sheep, and goats, is also affected by various infectious enteric diseases caused by pathogens such as *Rotavirus*, *E. coli*, *Salmonella* spp., *Clostridium perfringens*, and *Cryptosporidium*. These diarrheal diseases, commonly referred to as scours, pose significant challenges to economic productivity and animal welfare, leading to increased morbidity and mortality rates [117,118,119,120]. As in the swine and poultry industries, the use of antimicrobials to treat infectious diarrhea is increasingly restricted due to the emergence of AMR [121].

These enteric diseases are critical at two stages in the life of ruminants: during the transition period of the dams and the post-weaning period of their offspring [122,123,124]. In the transition period, which occurs from 3 weeks pre-calving until 3 weeks post-calving, important physiological, metabolic and nutritional changes take place. This is when most metabolic disorders occur, such as ketosis, fatty liver syndrome, milk fever, metritis, mastitis, retained fetal membranes or displaced abomasum, and immune function can be affected [122,123,124].

To meet their nutritional requirements in the transition stage, these animals are often fed high levels of starchy concentrates. However, the rapid fermentation of starch by the rumen microbiome can lower rumen pH and increase the risk of metabolic disorders. In this context, yeast postbiotics have been used to enhance ruminal fermentation by improving feed digestibility. An in vivo trial in cows supplemented with two different postbiotics based on yeast-derived active metabolites from 30 days pre-calving to two months into lactation reported an increase in colostral immunoglobulin concentration, improved milk performance with higher fat and protein yields, and greater persistence of the lactation curve throughout the production period [125]. Furthermore, this SCFP supplementation enhanced immune modulation and liver metabolic function while supporting energy-corrected milk yield [126].

In multiparous goats, the administration of yeast-derived active metabolites increased ruminal propionate concentration and fiber digestibility while reducing the partitioning of energy toward methane. These changes were associated with enhanced milk production, suggesting that postbiotics may improve the efficiency of nutrient utilization for lactation [127].

In ewes, administration of a combination of live *S. cerevisiae* with yeast postbiotics—rich in mannan-oligosaccharides and β-glucans—and selenium-enriched yeast improved energy status, milk yield and composition, and oxidative status, while simultaneously suppressing the mRNA expression of proinflammatory genes during the peripartum period [128].

The post-weaning stage in ruminants occurs when the young animal transitions from dependence on the dam and maternal milk to social and nutritional independence. This period is characterized by an immature and not fully developed immune system in the offspring, during which infectious diseases such as those aforementioned may arise [129]. Moreover, it represents a stressful and critical transition that affects the animal’s ability to adapt to the abrupt dietary shift and may influence the severity of subsequent production losses [130].

A potential strategy to enhance ruminant performance and immune function in the post-weaning phase is the use of postbiotics. In particular, postbiotics derived from *S. cerevisiae* have demonstrated multiple benefits when included in the diets of various animal species. In newly weaned calves, supplementation with SCFP supported rumen development, improved metabolic function and enhanced immune function [131]. In another trial, Holstein bull calves supplemented with SCFP exhibited improved ADG post-weaning, as well as increased feed efficiency, and required fewer treatments for bovine respiratory disease compared with untreated controls. Finally, SCFP administration improved fecal scores, reduced the number of days with diarrhea, and enhanced calf survival [132].

Dietary supplementation with yeast-derived peptides in lambs has been shown to mitigate the reduction in ADG and to attenuate intestinal damage associated with early weaning. Authors suggest that this beneficial effect may be due to changes in the abundance of the genera *Lactobacillus*, *Ruminococcaceae_UCG-014*, *Senegalimassilia*, and *Catenisphaera* in the gut microbiota [133].

### 4.4. Aquaculture Applications

In recent years, aquaculture has grown rapidly, and, driven by the substantial increase in the global population, this sector has assumed a significant role in the global food supply [134,135]. This is particularly evident in Southeast Asia, where aquaculture has been practiced for over four thousand years as a traditional economic activity and is now undergoing remarkable development, accounting for the production of approximately 87.5 million tons of aquatic animals [136]. However, this growth has been accompanied by the emergence and re-emergence of several infectious diseases, which represent a major limiting factor and, in some cases, lead to severe economic losses. Taking these considerations into account, new strategies are required to prevent and control diseases in aquatic species [137].

Recent research has increasingly focused on yeast-derived postbiotics due to their potential to enhance stress resilience in intensively farmed species. Cheng et al. recently investigated the effects of the administration of fermented metabolites obtained from *S. cerevisiae* and *Lactobacillus acidophilus* to hybrid grouper (*Epinephelus fuscoguttatus* ♀ × *Epinephelus lanceolatus* ♂) [138]. Their study demonstrated that dietary supplementation with postbiotics improved growth performance, intestinal morphology, antioxidant capacity, and non-specific immunity in hybrid grouper. Mechanistically, this work showed that postbiotics modulated gut microbiota composition by suppressing pathogenic bacteria and enriching beneficial taxa, thereby enhancing nutrient utilization and resilience to stress [138].

In white leg shrimp (*Litopenaeus vannamei*), administration of SCFP following a *Vibrio parahaemolyticus* challenge was associated with enhanced immune parameters. Although immune responses initially declined after pathogen exposure, they recovered to baseline levels along with the improvement of clinical signs of infection. Additionally, SCFP supplementation significantly improved survival rates compared with non-supplemented controls [139]. A similar study conducted in Pacific white shrimp (*Penaeus vannamei*) demonstrated improvements in growth performance and nutrient utilization, enhanced innate immunity, and strengthened resistance against *V. parahaemolyticus* [140]. Two postbiotics derived from the non-*Saccharomyces* yeast *Rhodotorula mucilaginosa*, inactivated by either heat treatment or sonication, were studied in *P. vannamei* challenged with *V. parahaemolyticus*. Results showed that dietary *R. mucilaginosa* supplementation significantly improved the disease resistance of shrimp against *V. parahaemolyticus* [141].

Finally, other species of great importance in aquaculture include channel catfish (*Ictalurus punctatus*) and Nile tilapia (*Oreochromis niloticus*). Using an in vivo approach, both species were fed with a fermented *S. cerevisiae* product and subsequently challenged with *Edwardsiella ictaluri* S97-773 or virulent *Aeromonas hydrophila* ML09-119 in catfish and *Streptococcus iniae* in Nile tilapia. The study demonstrated an increased survival of Nile tilapia following *S. iniae* infection, as well as modulation of the expression profiles of proinflammatory genes and antibodies [142]. However, in catfish, yeast supplementation produced more pronounced effects during shorter feeding periods of one week, resulting in increased survival against *E. ictaluri* and enhanced protection against *A. hydrophila* [142]. Researchers investigated the effects of SCFP on Nile tilapia and found that it enhanced growth performance and supported improved production efficiency in tilapia aquaculture [143].

**Table 1:** In vivo postbiotic evaluation studies in livestock animals.

Livestock Animal	Postbiotic Strain(s)	Type of Postbiotic	Pathogen	Postbiotic Dosage	Main Findings	References
Sows	*P. guilliermondii*	Inactivated yeast	ETEC	n.i.	Increased number of piglets born alive, earlier weaning of piglets, increased number of weaned piglets, lower mortality rate in piglets, piglets with greater weight gain and survival rate.	[97]
	*S. cerevisae*	SCFP	w/o inf.	2.0 kg/mT	Reduced diarrhea incidence and upregulated expression of tight junction proteins claudin-1 and occludin.	[98]
	*S. cerevisae*	Inactivated yeast	*E. coli* F4/F18	50 mL	Improved lactation efficiency and lower piglet mortality.	[99]
	*S. cerevisae*	Fermentation metabolites	w/o inf.	1.25 or 2.00 g/kg	Greater deposition of backfat in sows, increased weaning weight of piglets, decreased mortality and diarrhea index in piglets, lower glutathione peroxide and higher content of immunoglobulin A and malondialdehyde content in sows’ serum, increased lactose content and decreased secretory immunoglobulin A (sIgA) content in milk, higher immunoglobulin G (IgG) content, increased antioxidant capacity in sow placenta and content of transforming growth factor-β, and increased IgG and immunoglobulin M content in piglet serum.	[100]
	*S. cerevisae*	Bioactive metabolites	w/o inf.	1.25 or 2.00 g/kg	Increased chao1 index and α diversity of fecal microorganisms in sows during lactation, increased Actinobacteria and *Limosilactobacilli* in sows’ feces during pregnancy, and increased beneficial bacteria in piglet feces, thereby improving intestinal health.	[101]
	*S. cerevisae*	SCFP	ETEC	1.25 or 2.00 g/kg	Increased ADG, enhanced anti-oxidative capacity, decreased IL-2, IL-8 and INF-γ, during ETEC challenge: alleviated ETEC-induced ADG reduction, diarrhea, damages in intestinal permeability and morphology, and downregulation of tight junctions (claudin1 and occludin), alleviated ETEC-induced inflammation (decreased IL-6, TNF-α, INF-γ, and increased IL-4 and IL-10 in serum or jejunal mucosa, and increased serum IgG and mucosal sIgA).	[103]
	*P. kudriavzevii* FZ12	Autolyzed yeast	w/o inf.	0.5%	Diarrhea prevention, growth performance promotion in weaned piglet, enrichment of beneficial intestinal bacteria, especially the *Lactobacillus* species, important role in the growth and colonization of *Lactobacillus.*	[104]
	n.i.	Yeast cell wall and hydrolyzed yeast cells	w/o inf.	2.5 kg	Improved redox status, potential hepatoprotective properties and enhanced growth performance in weaned piglets.	[105]
Poultry	*S. cerevisae*	Yeast-derived mannan-rich fraction	w/o inf.	n.i.	Improved broiler performance, effective alternative to in-feed antibiotic growth promoters, and reduced environmental impact of poultry meat production.	[111]
	*S. cerevisae*	SCFP	w/o inf.	1.25 kg/MT	Improved feed conversion ratio, lowered levels of cholesterol and corticosterone, lower pathogenic and antibiotic-resistant bacteria, and improved humoral immunity.	[112]
	*S. cerevisae*	Enzymatically and heat-treated	w/o inf.	10^8^ *S. cerevisae*	Increased body weight, body weight gain, improved villus height, increased the numbers of *Lactobacillus* spp. in the cecum, and enhanced immune response and anti-inflammatory capacity.	[113]
	*S. cerevisae*	Fermentation-derived bioactive compounds	*N. enteritis*	1.6 mL/L	Improved performance, ameliorated pathology detriments associated with necrotic enteritis, and positive regulation of mRNA levels of key nutrient transporters during necrotic enteritis challenge.	[114]
	*S. cerevisae*	SCFP	*S. enteritidis*	2.5 lbs./ton	Reduced cecal *S. enteritidis* loads and a reduced number of birds testing positive for *S. enteritidis.*	[115]
	*S. cerevisae*	SCFP	w/o inf.	0.625 kg/MT	Reduced stress and improved feed conversion ratio.	[116]
Ruminants	n.i.	Yeast-derived active metabolites	w/o inf.	n.i.	Increased voluntary intake of dry matter, increased colostral immunoglobulin concentration, higher milk production, with high fat and protein yields and a higher persistence of the production curve throughout lactation.	[125]
	*S. cerevisae*	SCFP	w/o inf.	19 or 40 g/d	Improved lactation performance, improved metabolic status of dairy cows, reduced inflammation and enhanced liver metabolic function, resulting in greater milk fat, ECM, and FCM yield.	[126]
	n.i.	Yeast-derived active metabolites	w/o inf.	3.75 g/d	Increased energy efficiency for milk production and reduced CH_4_ emission per unit of milk edible product in late lactation.	[127]
	*S. cerevisae*	Combination of live *S. cerevisiae*, mannoproteins and β-glucans	w/o inf.	2 g/kg	Improved energy status, milk yield and some milk constituents, and oxidative status, suppression of mRNA levels of proinflammatory genes during the peripartum period.	[128]
	*S. cerevisae*	SCFP	w/o inf.	5 g/d	Enhanced rumen development, improved metabolic and immune function, and consequently improved future productivity in dairy cows.	[131]
	*S. cerevisae*	SCFP	w/o inf.	1 g/d	Improved average daily gain post-weaning, increased feed efficiency, reduced treatments for bovine respiratory disease, improved fecal scores, reduced number of days with diarrhea, and enhanced calf survival.	[132]
	n.i.	Yeast peptides	w/o inf.	5000 mg/kg	Reduced average daily gain and intestinal damage associated with early weaning due to changes in the abundance of genera *Lactobacillus*, *Ruminococcaceae_UCG-014*, *Senegalimassilia*, and *Catenisphaera* in the gut microbiota of lambs.	[133]
Aquaculture	*S. cerevisae*	Fermented derived compounds	w/o inf.	0.25, 0.75 or 2.00 mL/kg	Optimized growth performance, intestinal morphology, antioxidant capacity, and non-specific immunity, modulated gut microbiota composition by suppressing pathogenic bacteria and enriching beneficial taxa, thereby enhancing nutrient utilization and resilience to stress.	[138]
	*S. cerevisae*	SCFP	*V. parahaemolyticus*	0.45%	Enhanced the immune response and disease resistance of white leg shrimp against the pathogenic bacterium *V. parahaemolyticus* in *L. vannamei.*	[139]
	*S. cerevisae*	SCFP	*V. parahaemolyticus*	0.25%, 0.35%, or 0.45%	Improved growth performance and nutrient utilization, enhanced innate immunity, and strengthened resistance against *V. parahaemolyticus* in *P. vannamei.*	[140]
	*R. mucilaginosa*	Heat-killed or sonicated	*V. parahaemolyticus*	1 × 10^10^ cfu /kg	Improved the disease resistance of *P. vannamei* against *V. parahaemolyticus.*	[141]
	*S. cerevisae*	Fermented derived compounds	*S. iniae*, *E. ictaluri* or *A. hydrophila*	0.1% or 0.4%	Increased survival of Nile tilapia following *S. iniae* infection, modulation of the expression profiles of proinflammatory genes and antibodies, and increased survival against *E. ictaluri* and enhanced protection against *A. hydrophila* in catfish.	[142]
	*S. cerevisae*	SCFP	w/o inf.	2 g/kg, 4 g/kg, or 6 g/kg.	Enhanced growth performance and supported improved production efficiency in Nile tilapia aquaculture.	[143]

n.i.: not indicated, w/o inf.: without infection.

## 5. Challenges and Knowledge Gaps

Despite the promising findings summarized above, several challenges remain regarding the use of yeast-derived postbiotics. One of the key limitations is the absence of a consensus on the definition and criteria used to determine whether a given compound or preparation can be classified as a postbiotic. Although ISAPP has defined the term *postbiotic*, the relatively recent nature of this definition has led to confusion within the food, medical, and veterinary sectors, impacting both the production and application of these biotherapeutic agents [31,144]. Such ambiguities may result in inconsistencies in postbiotic preparation methods, potentially affecting the characteristics, as well as its scalability for industrial production [32,59,60,144]. This underscores the need to establish standardized protocols for postbiotic preparation, ideally developed by ISAPP or other relevant organizations in collaboration with panels of experts [144]. Moreover, consistent and accurate use of terminology in all scientific communications, along with clear guidelines for specific contexts—such as food labeling and clinical trials—is strongly recommended [31,144]. Several countries, including Canada, China, and Australia, have taken steps toward establishing regulatory approaches for postbiotic use. However, the current global regulatory landscape remains heterogeneous [145,146] and existing regulatory frameworks designed for probiotics are not appropriate for postbiotics. This situation has resulted in a regulatory vacuum, thereby facilitating the commercialization and development of postbiotic-based products beyond the minimal requirement of demonstrating the non-toxicity of their components [147].

Although several postbiotic strains have been investigated, most studies have focused on a limited number of well-established strains, leaving many species insufficiently characterized [148]. With respect to yeast strains, significant knowledge gaps remain. Current postbiotic research has largely focused on *S. cerevisiae* and *S. cerevisiae* var. *Boulardii*, whereas a limited number of studies have explored the postbiotic potential of other yeast species, such as *Wickerhamomyces anomalus*, *R. mucilaginosa*, *Pichia fermentans* or *K. marxianus* [55,141,149], despite their demonstrated ability to produce a wide range of metabolic compounds that may improve shelf-life, sensory properties, and safety and have potential health benefits [150].

This limited understanding of postbiotic species is particularly important because host responses can be species-specific, as different bacterial species and strains produce structurally distinct MAMPs, including unique lipoteichoic acids, peptidoglycans, and exopolysaccharides [148]. These MAMPs interact with PRRs, such as TLRs and NOD-like receptors (NLRs), with varying affinities, resulting in distinct signaling outcomes [151].

Given the relatively recent emergence of this field, there is a notable lack of studies evaluating the long-term effects of postbiotic administration. While short-term clinical trials, animal models, and in vitro experiments have provided valuable insights, extended investigations in applied or field-based settings remain scarce. This highlights a critical need for long-term studies to better understand the sustained impact, safety, and efficacy of postbiotics over time, particularly under practical conditions relevant to livestock or human applications [152,153].

Inclusion rates of postbiotics in farm animal diets present a challenge, as certain postbiotics require relatively high doses to confer measurable health benefits, depending on the specific product and its processing [154]. This reinforces the concept of dose dependence for structurally bioactive postbiotics, such as β-glucans and mannoproteins, and highlights the need for relatively high levels of these compounds in postbiotic formulations [155]. Moreover, considering their dose-dependent nature, the misuse of postbiotics at levels exceeding the dosage recommended by the manufacturer may lead to immune overstimulation and, consequently, a lack of improvement in overall animal health [156]. Another limitation is the duration of supplementation, as yeast-derived postbiotics require prolonged and consistent administration to achieve measurable effects. Studies indicate that these postbiotics are most effective when provided continuously or over extended periods [45,126,157,158].

Finally, these challenges have a direct impact on the livestock industry, which faces several limitations. In terms of biological and efficacy considerations, no single yeast strain is universally effective across all animal species, as the same strain may elicit different responses in different hosts [159]. Moreover, information is lacking regarding the optimal life stage at which yeast postbiotics should be administered [159]. In addition, the absence of global regulatory policies complicates the commercialization and export of these products, thereby constraining their broader use in livestock supplementation [146].

Altogether, the available evidence indicates that significant efforts are still needed to clarify terminology, establish robust regulatory frameworks, and expand research on novel yeast strains, postbiotic components, and their stability. In our view, particular attention should be given to determining optimal dosage, treatment duration, and the effects of prolonged administration, as these aspects are critical in fully realizing the potential of yeast postbiotics in livestock production.

## 6. Future Directions

There is a clear need for further investigation in the field of postbiotics and more specifically yeast-derived postbiotics, with omics-based approaches representing one of the most promising directions for future research. In this context, a novel approach known as *probiogenomics* has emerged, encompassing the application of high-throughput techniques such as genomics, transcriptomics, proteomics, and metabolomics. These methodologies provide a valuable framework for predicting strain functionality and enabling the rational selection of novel, previously uncharacterized strains. Probiogenomics may facilitate the screening of multiple strains to identify potentially relevant traits and support an in-depth characterization of microbial physiology, functionality, and mechanisms of action. Furthermore, genomic screening plays a crucial role in ensuring safety by confirming the absence of undesirable features, including antibiotic resistance genes or virulence factors [160].

Considering the rapid expansion of artificial intelligence (AI) in life sciences, the field of postbiotics has begun to explore AI as an advanced approach to identifying potential therapeutic compounds and analyzing their effects on metabolic pathways [161]. This integration can facilitate the development of novel and personalized treatments, potentially reducing the need for extensive clinical trials and accelerating approval processes. However, several challenges must be addressed to fully realize these benefits, including standardization of protocols, rigorous quality control, deeper understanding of postbiotic mechanisms and the development of sophisticated algorithms and high-quality datasets to accurately predict postbiotic effects [161,162]. Additionally, ethical and regulatory considerations must also be carefully evaluated to ensure that AI applications in postbiotic research are reliable, unbiased, and accountable [162]. Despite these limitations, integrating AI holds great promise for advancing our understanding of postbiotics, discovering new strains, and developing innovative strategies to improve livestock health and manage a wide range of pathological conditions [161,162].

Another emerging strategy in animal farming is precision livestock management, which integrates sensors, artificial intelligence, and data analytics to monitor animal health, feed intake, and performance in real time, thereby enabling targeted and timely interventions. This approach not only optimizes nutrient utilization but also enhances overall production efficiency by reducing feed costs while maximizing growth rates and milk yield. Within this framework, the delivery of postbiotics has gained increasing attention, as these compounds can improve gut health, immune responses, and growth performance in livestock while avoiding the risks associated with the administration of live microorganisms [163].

In the context of AMR, a novel and promising strategy is the use of postbiotics as adjuvants to antibiotic therapy, with the potential to reduce antibiotic dosages and limit the selection of resistant strains [164,165]. Several in vitro studies have explored the combination of these compounds, reporting enhanced antibacterial effects [164,165]. Nevertheless, further research is required, including well-designed clinical trials and real-world implementation studies, to establish their efficacy, optimal dosing, safety profiles, and appropriate monitoring criteria before their routine incorporation into antimicrobial stewardship programs [165].

Finally, researchers are exploring the use of postbiotics in combination with vaccines, primarily as immune-modulating, “adjuvant-like” agents capable of enhancing vaccine-induced responses and potentially reducing the required antigen or adjuvant doses [166]. However, most available evidence is derived from small-scale clinical studies, and large, well-designed randomized trials are required to determine the most effective postbiotic candidates, optimal dosing regimens, timing of administration, and compatible vaccine platforms, as well as to confirm long-term safety [167,168].

Overall, the findings reported to date indicate that further research is necessary to drive continued progress in this field. Nonetheless, given the tools and methodologies currently available, future developments are highly promising. From our perspective, continued investigation is essential, as furthering knowledge in this area has considerable potential to enhance health outcomes and improve the management of gastrointestinal infections.

## 7. Conclusions

In conclusion, yeast postbiotics in livestock represent a promising and rapidly emerging field of research due to their multifactorial modes of action. Based on the evidence analyzed in this review, we consider that this biological complexity underpins their broad spectrum of beneficial effects and positions yeast postbiotics as valuable tools for disease mitigation and overall health improvement. In livestock production, these postbiotics appear particularly effective in controlling enteric diseases across multiple animal species, while also supporting efforts to mitigate antimicrobial resistance within a One Health framework.

At the same time, we consider that several critical aspects require further research and clarification to consolidate their practical implementation. A greater consensus on terminology, the development of robust regulatory frameworks, and more standardized experimental approaches are necessary to strengthen comparability among studies. Moreover, industrial scalability, long-term in vivo validation, and the determination of optimal dosage and treatment duration are, in our view, essential steps to fully unlock the practical potential of yeast postbiotics in livestock production.

## Data Availability

No new data were created or analyzed in this study.
